# Correlation of plasma and urine Wnt5A with the disease activity and cutaneous lesion severity in patients with systemic lupus erythematosus

**DOI:** 10.1007/s12026-021-09253-w

**Published:** 2021-12-03

**Authors:** Shuhong Chi, Jing Xue, Xiaodong Chen, Xiaoming Liu, Yanhong Ji

**Affiliations:** 1 Department of Pathogenic Biology and Immunology, School of Basic Medical Sciences, Xi’an Jiaotong University Health Science Centre, No. 76 Yanta West Road, Xi’an, 710061 Shanxi China; 2 Department of Rheumatology, General Hospital of Ningxia Medical University, Yinchuan, 750004 Ningxia China; 3 Human Stem Cell Institute, General Hospital of Ningxia Medical University, Yinchuan, 750004 Ningxia China; 4 Department of Anatomy and Cell Biology, Carver College of Medicine, The University of Iowa, 51 Newton Road, Iowa City, IA 52242 USA

**Keywords:** Wnt5A, Systemic lupus erythematosus, Cutaneous lesions, Biomarker

## Abstract

Reliable noninvasive biomarkers are needed to accurately assess disease activity and prognosis in patients with systemic lupus erythematosus (SLE). The purpose of this study was to investigate the clinical relevance of Wnt5A with disease activity and severity with cutaneous involvement in particular in SLE patients; its concentrations in plasma and urine were examined and analyzed. In the cross-sectional study, the clinical relevance of Wnt5A protein was evaluated in both plasma and urine of SLE patients and healthy cohorts using commercial enzyme-linked immunosorbent assays (ELISA). Significantly, more abundances of Wnt5A protein were determined in both of plasmas and urines of SLE patients compared to healthy cohorts (*p* < 0.0001), which were even higher in active disease (AD) SLE patients relative to low disease activity (LDA) SLE patients (*p* < 0.0001). Meanwhile, the ROC curve analysis demonstrated that the plasma and urine Wnt5A were potential candidate biomarkers for identifying the disease activity and severity in SLE patients. The discriminant function analysis further revealed that the plasma and urine Wnt5A were separated and distinct for AD SLE patients and healthy controls. In consistence, the disease severity was correlated with the plasma and urine Wnt5A as ascertained by CLASI activity score and the prevalence of serositis in SLE patients. These results suggest that Wnt5A, as a summary measure for different inflammatory processes, could be a potential biomarker for accessing the disease activity, and a noninvasive biomarker for evaluating the disease severity in terms of cutaneous involvement in SLE patients.

**Supplementary Information:**

The online version contains supplementary material available at 10.1007/s12026-021-09253-w.

## Introduction

Systemic lupus erythematosus (SLE) is a chronic inflammatory disease, which is developed in genetically susceptible individuals in response to environmental factors [1, 2]. It belongs to an autoimmune rheumatic disease characterized by the production of various autoantibodies and cytokines, such as antinuclear antibodies (ANA) and interferon-α leading to systemic inflammation and multiple organ damage and dysfunction. As the etiology and pathogenesis underlying its development remain unclear, SLE is still associated with high morbidity and mortality. However, the clinical and paraclinical diagnostic tools to assess disease activity and severity of organ involvement are inadequate, and noninvasive accessible biomarkers are also required for SLE.

In general, the disease severity of SLE is accessed by evaluating symptoms that partly attributed to characteristic clinical findings in the skin, joints, kidneys, and central nervous system, as well as serological parameters such as ANA, cytokine, and immune complex deposition [3]. However, the various clinical symptoms do not always occur simultaneously and may develop at any stage of the diseases. In the early stages, physicians from various disciplines often propose several differential diagnoses, or identify only one aspect of the disease without recognizing the symptoms as part of SLE [4, 5]. As being noted above, serology is of particular value in situations where clinical expression of the disease is incomplete, when the presence of a particular ANA profile can be diagnostic. In addition, ANA can also be found in organ-specific autoimmune diseases and in other clinical settings such as infection and lymphoproliferative disorders. Equally noteworthy, the frequency of ANA in normal individuals is usually higher in woman, and is increased with age that more than 25% of women are ANA-positive at an age of 60 years and older. Such a lack of specificity of ANA for SLE has also been observed in other conventional parameters such as double-stranded DNA (dsDNA), and concentrations of complements C3 and C4 [6, 7]; this creates a range of clinical dilemmas challenging both patients and practitioner, which thus led to search of other reliable biomarkers for accessing disease activity and severity of organ involvement.

A compelling body of studies recently demonstrated that the Wnt signaling is implicated in the pathogenesis of many diseases. The Wnt signaling can be characterized as either the β-catenin-dependent canonical Wnt pathway or the β-catenin-independent noncanonical Wnt pathways which includes at least the planar cell polarity (PCP) pathway and Wnt/calcium pathway [8]. In this context, the Wnt5A is a representative ligand activating noncanonical Wnt signaling [9]. Owing to the Wnt5A which has recently emerged as a macrophage that triggers inflammation, there are several studies supporting significant progress which has been made in investigating the role of Wnt5A in various chronic inflammatory/autoimmune rheumatic diseases, including sepsis, ankylosing spondylitis (AS), rheumatoid arthritis (RA), and atherosclerosis [10]. For instance, increased transcripts of Wnt5A and its receptor Fzd5 were observed in RA synovial tissues, and blocking the Wnt5A/Fzd5 signaling reduced the rheumatoid synoviocyte activation [10]. Interestingly, a further study found that the pro-inflammatory cytokine IL-6 could induce Wnt5A which had implicated in synovial fibroblast hypertrophy in RA [11]. Similarly, we recently showed that plasma Wnt5A could be a biomarker of disease activity in patients with RA-associated interstitial pneumonia (RA-ILD) [12]. Moreover, large-scale gene correlation network analysis has shown hsa_circ_0001449 regulates SLE progression by regulating metabolic pathways (80.9%) and the noncanonical Wnt signaling (4.3%) [13]. These studies suggest that Wnt5A-mediated noncanonical Wnt signaling may have a clinical implication in the development of SLE patients; therefore, an assessment of Wnt5A may offer clinical significances for identifying and monitoring SLE.

Here, the concentration of Wnt5A protein in both plasmas and urines of clinically diagnosed SLE patients from a single center was examined and analyzed by comparing with other clinical indexes. The results demonstrated a strong correlation of the plasma and urine Wnt5A protein with the disease severity in SLE patients, particularly those with active disease (AD).

## Materials and methods

### Ethics statement

Human blood and urine samples were collected with a protocol approved by the Ethics Committee for the Conduct of Human Research at General Hospital of Ningxia Medical University (NXMU-GH-2019-487). Written consent was obtained from every individual for collecting blood and urine samples and publishing the data according to the Ethics Committee for the Conduct of Human Research protocol. All participants were older than age of 21 years old. The PI of this study maintains human research records, including signed and dated consent documents, for ten (10) years after their acquisition. The Ethics Committee for the Conduct of Human Research at Ningxia Medical University approved the consent procedure for this study (NXMU-GH-2019-487).

### Sample size calculation

The sample size was calculated based on the formula for correlation study [14] and G*power (version 3.1.9) (G*power Software, the University of Dusseldorf, Germany) as stated below: *α* (2 tailed) = 0.05 (threshold probability for rejecting the null hypothesis); *β* = 0.20 (probability of failing to reject the null hypothesis under the alternative hypothesis); effect size = 0.5; total sample size = 128.

### Human subjects

Blood samples of 115 consecutive SLE patients (80 females and 35 males) and urine samples of 128 consecutive SLE patients (81 females and 47 males) were collected from the outpatient rheumatology clinics of General Hospital of Ningxia Medical University from July to December in year 2019. The American College of Rheumatology (ACR) criteria were used to diagnose patients with SLE [15, 16], and the disease activity was defined according to SLE Disease Activity Index (SLEDAI) criteria [17, 18]. A patient with SLEDAI ≥ 10 was defined as AD SLE, and SLEDAI < 10 was defined as low disease activity (LDA) SLE [19]. All the SLE patients included in this study received standard-of-care pharmacological treatment that did not include biological agents. Plasmas and urines of 82 gender- and age-matched healthy individuals were also collected. These healthy control cohorts were recruited from those who had undergone comprehensive medical screening at the General Hospital of Ningxia Medical University and who had no history of chronic diseases and no family history of autoimmune diseases. The demographics of individuals involved in this study are outlined in Suppl. Table S1.

### Cutaneous disease activity score and damage score

**Fig. 1:**
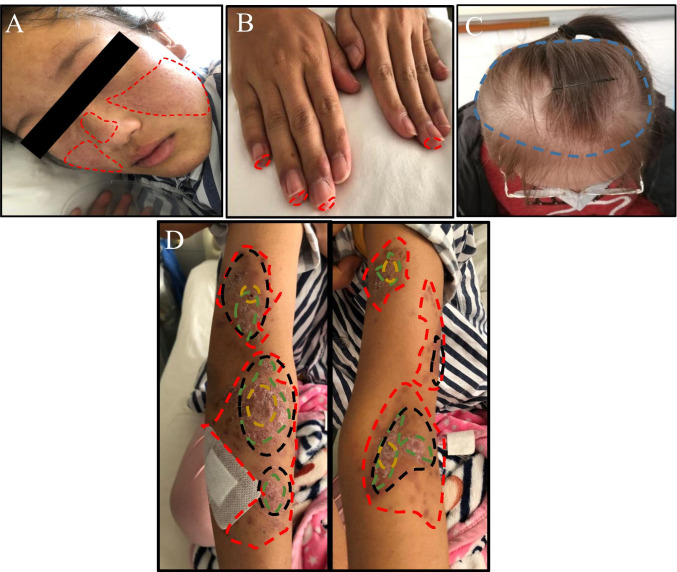
Representative images of cutaneous lesions with various severity of SLE and scoring criteria. **A**–**D** An example of the cutaneous lesions in the study represented images of a 23-year-old female. **A** Erythema was graded as score 3 on the both cheeks and score 1 on the nose, thus giving a total score of 4 for erythema in the face. **B** Erythema was graded as score 1 on the four fingertips of the both hands. **C** Alopecia was graded as score 1 in hairy areas of the scalp. **D** Erythema, scale, scarring, and dyspigmentation were graded as respective scores of 3, 1, 1, and 1 on both arms, thus giving a total score of 6 in arms. In total, CLASI activity score of 10 and CLASI damage score of 2 reflect the extent of cutaneous lesions. Red: erythema; green: scale; blue: alopecia; yellow: scarring; black: dyspigmentation

Cutaneous disease activity and damage were evaluated using the Cutaneous Lupus Erythematous Disease Area and Severity Index (CLASI) [20]. The total CLASI activity score is the arithmetic sum of each of the individual scores of items in a given cutaneous activity field. Lesions are rated for erythema and scale/hypertrophy based on the regions affected. Mucous membrane involvement and alopecia are also assessed (Suppl. Table S2) (Fig. 1A–D). The total CLASI damage score is the arithmetic sum of the items rated regionally for damage caused by dyspigmentation, scarring/atrophy/panniculitis, and scarring of the scalp (Suppl. Table S2) (Fig. 1A–D). Scores can range from 0 to 70 for CLASI activity and 0 to 70 for CLASI damage, with higher scores denoting greater disease activity or damage (Suppl. Table S2).

### Detection of plasma and urine Wnt5A by enzyme-linked immunosorbent assays

The concentrations of Wnt5A protein in plasma and urine were measured using commercially available enzyme-linked immunosorbent assays (ELISA) kit per manufacturer’s instruction (assay range: 0.25–8.00 ng/mL). The ELISA kit for Wnt5A was a product of ML Bio Inc. (Shanghai, China). For detection of Wnt5A protein, the plasma and urine were directly detected with stock suspension, and their concentrations were presented as nanograms per milliliter through a standard curve. The standard curve was generated by plotting the average O.D. (450 nm) obtained for each of the six standard concentrations on the vertical (*X*) versus the corresponding concentration on the horizontal (*Y*) axis, and all O.D. values were subtracted by the mean of the blank value before result interpretation. Values outside the range of standard curves were generally nonlinear, which could lead to incorrectly extrapolated values. Samples that generated a value greater than standard ranges were further diluted for repetitive measurements. If a value fell below the range of the assay, the sample was considered to be undetectable.

### Statistical analysis

All laboratory data were entered into and extracted from PRISM (version 8.0) (GraphPad Software, La Jolla, CA, USA) and/or Excel (version 2016) (Microsoft Software, Redmond, WA, USA) and/or SPSS for Windows (version 26.0) (SPSS Inc., Chicago, IL, USA). One-way ANOVA or Kruskal-Wallis test was employed for comparisons of more than two groups, and the *t*-test was conducted for comparison between two groups. ROC (receiver operator characteristic) curve was generated for determining an optimal cutoff value and validity of certain variable. The association between qualitative variables was evaluated by Spearman correlation. Data was presented as the mean standard error of mean (SEM) or mean ± standard deviation (SD). A value of less than 0.05 was considered statistically significant, *< 0.05; **< 0.01; and ***< 0.0001.

## Results

### Elevated Wnt5A protein in plasmas and urines of SLE patients

**Fig. 2:**
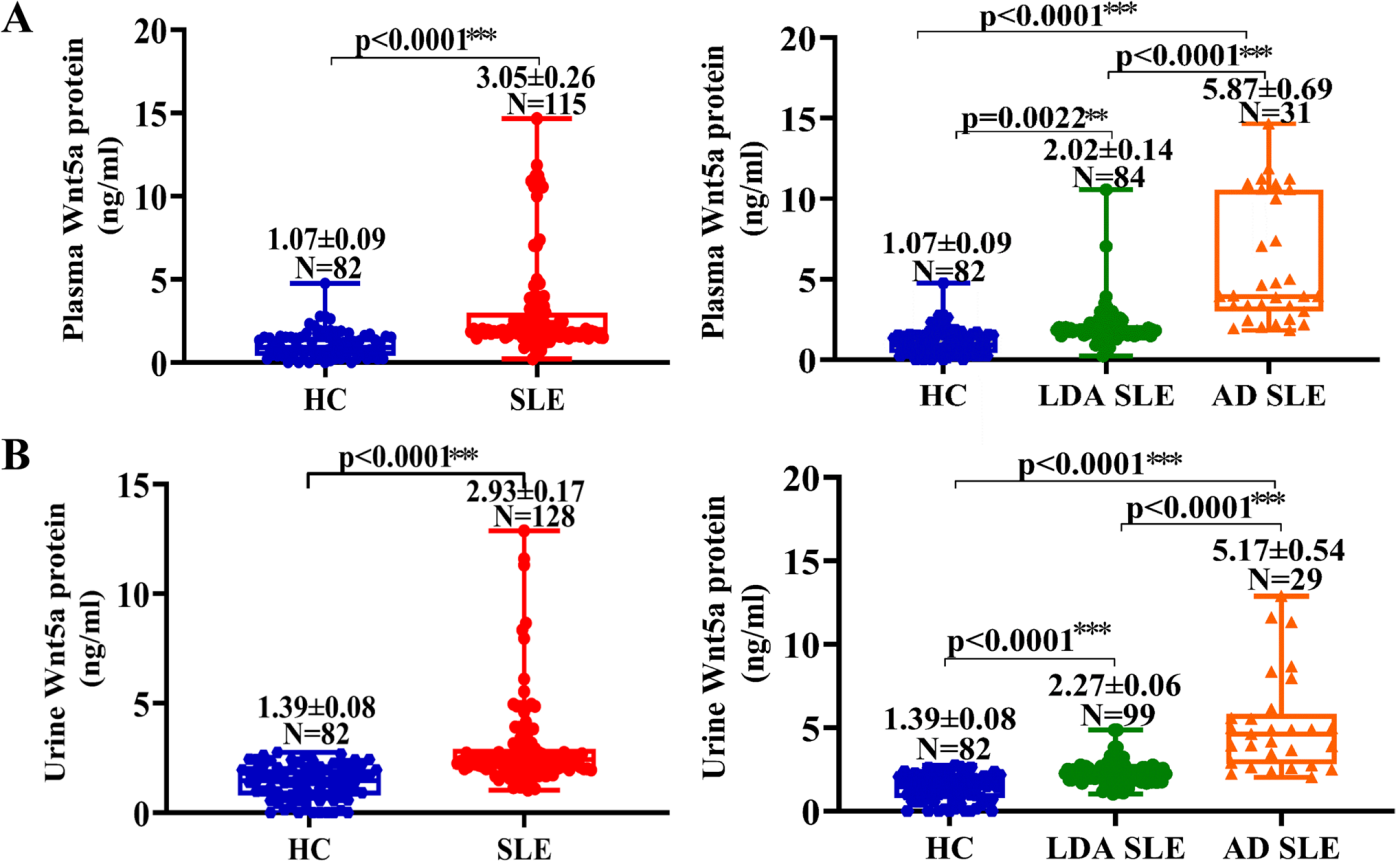
The concentrations of Wnt5A protein in plasma and urine of healthy individuals and SLE patients. **A** The plasma concentration of Wnt5A protein in healthy subjects and SLE patients. Statistical differences were found between healthy individuals and SLE patients (left panel, *p* < 0.0001), healthy individuals and LDA SLE patients (right panel, *p* = 0.0022), healthy individuals and AD SLE patients (right panel, *p* < 0.0001), and LDA and AD SLE patients (right panel, *p* < 0.0001). More abundant plasma Wnt5A protein was detected in AD SLE patients relative to healthy individuals and LDA SLE patients, and the highest concentration of plasma Wnt5A protein was determined in AD SLE patients. **B** The urine concentration of Wnt5A protein in healthy subjects and SLE patients. Statistical differences were found between healthy individuals and SLE patients (left panel, *p* < 0.0001), healthy individuals and LDA SLE patients (right panel, *p* < 0.0001), healthy individuals and AD SLE patients (right panel, *p* < 0.0001), and LDA and AD SLE patients (right panel, *p* < 0.0001). Like what is seen in plasma, more abundant urineWnt5A protein was detected in AD SLE patients relative to healthy individuals and LDA SLE patients, and the highest concentration of urine Wnt5A protein was determined in AD SLE patients. **p < 0.01; ***p < 0.001. Data represent the mean ± SEM in each group.

To determine whether Wnt5A protein was correlated with SLE activity, both plasma and urine concentrations of Wnt5A were evaluated in SLE patients with LDA and AD and healthy subjects. Notably, a significantly abundant plasma Wnt5A protein was found in SLE patients (3.05 ± 0.26 ng/mL) than that in healthy subjects (1.07 ± 0.09 ng/mL) (*p* < 0.0001) (Fig. 2A). More importantly, a strikingly higher concentration of plasma Wnt5A was found in SLE patients with AD (5.78 ± 0.69 ng/mL) in comparison with those with LDA (2.02 ± 0.14 ng/mL) (*p* < 0.0001) (Fig. 2A). Similar to plasma Wnt5A, a significantly higher concentration of urine Wnt5A protein was determined in SLE patients (2.93 ± 0.17 ng/mL) relative to healthy individuals (1.39 ± 0.08 ng/mL) (*p* < 0.0001) (Fig. 2B). In addition, even higher urine Wnt5A concentration was determined in AD SLE patients (5.17 ± 0.54 ng/mL) compared with LDA SLE patients (2.27 ± 0.06 ng/mL) (*p* < 0.0001) (Fig. 2B).

### Correlations between Wnt5A concentrations and disease activity index

**Fig. 3:**
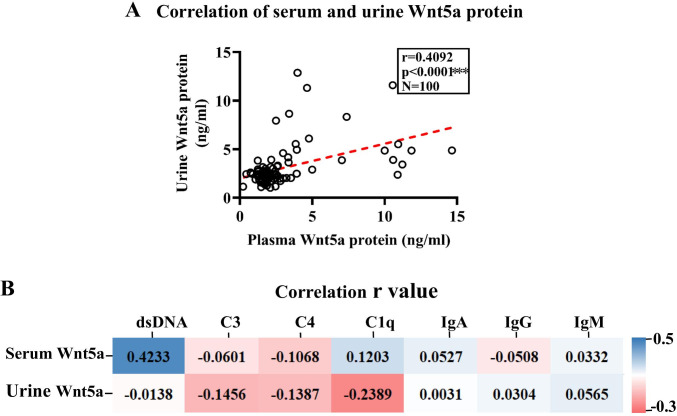
Correlations of Wnt5A protein and other risk factor in SLE patients. **A** Correlation between Wnt5A protein in plasma and urine. **B** Correlation matrix heatmap of plasma and urine Wnt5A and the clinical other serological biomarkers dsDNA, C3, C4, IgA, IgM, and IgG. Spearman and values are displayed on each graph. A value was determined by the two-tailed Pearson correlation test. *r*, Spearman’s correlation coefficient; C3, complement C3; C4, complement C4; dsDNA, double-stranded DNA; IgA, immunoglobulin A; IgG, immunoglobulin G; IgM, immunoglobulin M

Above data showed that Wnt5A protein was abundant in both plasma and urine of SLE patients compared with those of healthy subjects; the correlation of Wnt5A protein in plasma and urine was analyzed. Expectedly, there was an association between plasma and urine in SLE patients determined for Wnt5A (*r* = 0.4092, *p* < 0.0001, *N* = 100) (Fig. 3A). Interestingly, association analysis of plasma or urine Wnt5A and clinical other serological biomarkers showed the plasma Wnt5A was positively correlated with SLE serological marker dsDNA (*r* = 0.4233, *p* < 0.0001) and the urine Wnt5A was positively correlated with anti-C1q antibody (*r* = − 0.2389, *p* = 0.0033) (Fig. 3B). In addition, the correlation coefficients between plasma Wnt5A and C3, Wnt5A and C4, Wnt5A and anti-C1q, Wnt5A and IgA, Wnt5A and IgG, and Wnt5A and IgM were *r* = − 0.0601 (*p* = 0.2618), *r* = − 0.01068 (*p* = 0.1279), *r* = 0.1203 (*p* = 0.1165), *r* = 0.0527 (*p* = 0.2877), *r* = − 0.0508 (*p* = 0.2877), and *r* = 0.0332 (*p* = 0.3713), respectively (Fig. 3B); the correlation coefficients between urine Wnt5A and dsDNA, Wnt5A and C3, Wnt5A and C4, Wnt5A and IgA, Wnt5A and IgG, and Wnt5A and IgM were *r* = − 0.0138 (*p* = 0.2382), *r* = − 0.1456 (*p* = 0.0506), *r* = − 0.1387(*p* = 0.0593), *r* = 0.0031 (*p* = 0.4860), *r* = − 0.0304 (*p* = 0.3663), and *r* = 0.0565 (*p* = 0.2632), respectively (Fig. 3B).

### Predictive usefulness of plasma and urine Wnt5A as biomarkers in active SLE

**Fig. 4:**
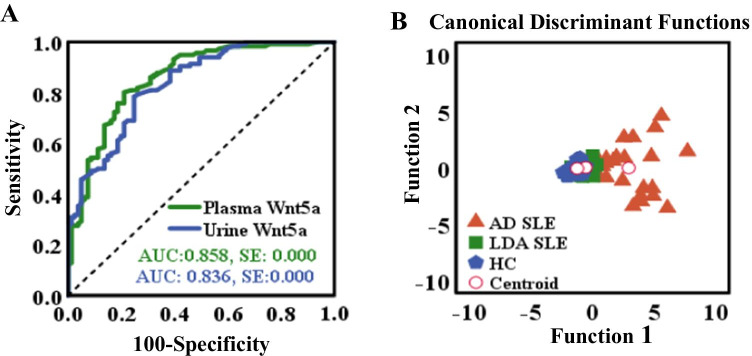
Evaluation of plasma and urine Wnt5A concentrations as potential biomarkers of active SLE. **A** Areas under the receiver characteristic (ROC) curve (AUCs) for prediction models discriminating SLE patients and healthy individuals. ROC curves are shown for plasma Wnta5A protein (green line), urine Wnt5A protein (blue line). **B** Discriminant analysis using plasma and urine Wnt5A levels to classify AD SLE patients (orange triangles), LDA SLE patients (green squares), and healthy individuals with no personal or family history of autoimmunity (blue diamonds). Two canonical discriminant functions, function 1 and function 2, were generated based on their individual standardized coefficients. There is clear discrimination among 3 groups, and the model predicts group membership with 79.5% accuracy. Red circles represent the group centroid. LDA, low disease activity; AD, active disease

In order to evaluate the significance of Wnt5A in clinical settings, we analyzed the sensitivities and specificities of plasma and urine Wnt5A for the identification of patients with SLE. The ROC curve showed that Wnt5A (Fig. 4A), particularly the plasma Wnt5A, was considered a better positive biomarker than negative in SLE with higher sensitivity (Fig. 4A). The area under curve (AUC) for plasma Wnt5A was 0.858 (SE: 0.000; range: 0.805–0.912; threshold: 1.5758) (Fig. 4A), and the AUC for urine Wnt5A was 0.836 (SE: 0.000; range: 0.780–0.891; threshold: 1.9834). Furthermore, the data were also subjected to multivariate statistical analyses to establish whether plasma and urine Wnt5A can be used to distinguish the AD SLE and LDA SLE groups. Discriminant function analysis was limited to them and included 25 AD SLE patients, 76 LDA SLE patients, and 26 healthy cohorts. This analysis revealed plasma and urine Wnt5A were separated and distinct only for the AD SLE patients and healthy controls (*p* = 0.000), while the separation of LDA SLE patients was less reliable (*p* = 0.687). The model predicted group membership based on these discriminant functions with an overall accuracy of 79.5%. These results may imply that both plasma and urine Wnt5A may be biomarkers for identification of disease activity in SLE patients, particularly those with AD SLE patients.

### Plasma and urine Wnt5A protein correlates the disease progression in SLE patients

**Fig. 5:**
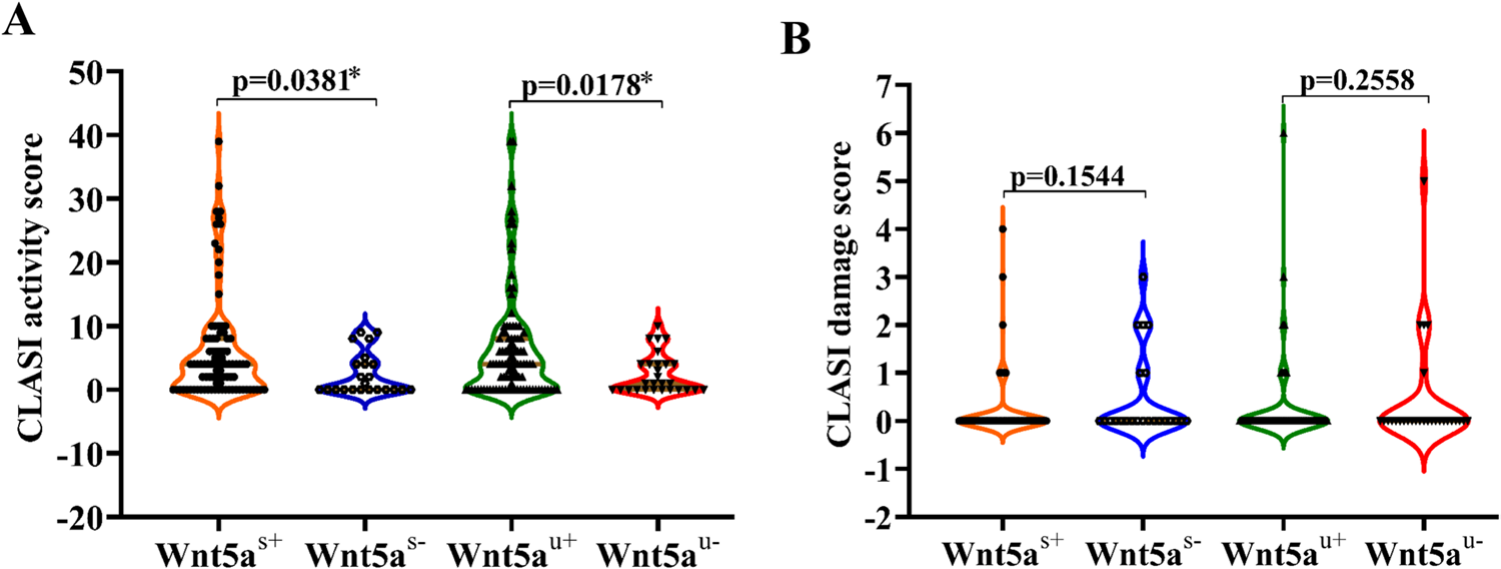
Association of plasma and urine Wnt5A protein with the severity of SLE patients. **A** The median CLASI activity score is shown according to plasma and urine Wnt5A protein status. Statistical differences were found between Wn5A^p+^ and Wn5A^p−^ patients (*p* = 0.0381), and Wnt5A^u+^ and Wnt5A^u−^ patients (*p* = 0.0178). The higher CLASI activity score was observed in plasma- and urine-positive Wnt5A relative to Wnt5A-negative patients. **B** The median CLASI damage score is shown according to plasma and urine Wnt5A protein status. No statistical differences were detected between Wn5A^p+^ and Wn5A^p−^ patients (*p* = 0.1544), and Wnt5A^u+^ and Wnt5A^u−^ patients (*p* = 0.2588). The brown line depicts the median, and the dotted line shows p25-p75. Wnt5A^p+^, plasma-positive Wnt5A protein; Wnt5A^p−^, plasma-negative Wnt5A protein; Wnt5A^u+^, urine-positive Wnt5A protein; Wnt5A^u−^, urine-negative Wnt5A protein

Next, we determined whether plasma or urine Wnt5A protein was correlated with the progression of disease severity; the Wnt5A patterns were first classified. As determined using a ROC threshold, 80.0% (92/115) of plasma Wnt5A and 78.1% (100/128) of urine Wnt5A were positive (Table 1). Of note, the respective prevalence of serositis in SLE patients with plasma-positive Wnt5A protein (Wnt5A^pp+^) and plasma-negative Wnt5A protein (Wnt5A^pp−^) were 28.3% and 4.3%, and 19.0% and 7.1% in SLE patients with urine-positive Wnt5A protein (Wnt5A^u+^) and urine-negative Wnt5A protein (Wnt5A^u−^). Suggesting that the prevalence of serositis was significantly associated with plasma- and urine-positive Wnt5A protein (*p* = 0.000, *p* = 0.000) (Table 1). Furthermore, CLASI activity score analysis revealed that the abundance of Wnt5A protein was associated with the activity score of CLASI. The CLASI activity score in Wnt5A^p+^ patients was higher compared to that in Wnt5A^p−^ patients (median: 4 versus 0.5; *p* = 0.0381, Fig. 5A). Equally important, the score was also significantly higher in Wnt5A^u+^ patients relative to Wnt5A^u−^ patients (median: 4 versus 1; *p* = 0.0178, Fig. 5A). But CLASI damage score showed that no difference was found between Wnt5A^p+^ and Wnt5A^p−^ patients, and Wnt5A^u+^ and Wnt5A^u−^ patients (Fig. 5B). Unlike what were seen in the prevalence of serositis and CLASI activity score, no difference in the prevalence of renal disorder, musculoskeletal, hematological, and neuropsychiatric was detected between Wnt5A^p+^ and Wnt5A^p−^ patients, and Wnt5A^u+^ and Wnt5A^u−^ patients. These results thus suggest that both plasma and urine Wnt5A protein may be biomarker candidate for evaluating severity of organ involvement, especially with regard to a cutaneous involvement.

**Table 1:** Clinical manifestations of SLE patients according to Wnt5a protein status

Clinical manifestations	Wnt5a^p+^ (*n* = 92)	Wnt5a^p−^ (*n* = 23)	*p*-value	Wnt5a^u+^ (*n* = 100)	Wnt5a^u−^ (*n* = 28)	*p*-value
Serositis (*n*, %)	26 (28.3%)	1 (4.3%)	0.000	19 (19.0%)	2 (7.1%)	0.000
Renal disorder (*n*, %)	45 (48.9%)	10 (43.5%)	0.641	56 (56.0%)	14 (50.0%)	0.573
Musculoskeletal (*n*, %)	40 (43.5%)	10 (43.5%)	1.000	44 (44.0%)	11 (39.3%)	0.656
Hematological (*n*, %)	90 (97.8%)	21 (91.3%)	0.127	93 (93.0%)	26 (92.9%)	0.979
Neuropsychiatric (*n*, %)	22 (23.9%)	5 (21.7%)	0.826	22 (22.0%)	7 (25.0%)	0.737
Cutaneous (*n*, %)	65 (70.6%)	11 (47.8%)	0.038	73 (73.0%)	12 (42.9%)	0.003

*Wnt5a*^*p+*^, plasma-positive Wnt5a protein; *Wnt5a*^*p−*^, plasma-negative Wnt5a protein; *Wnt5a*^*u+*^, urine-positive Wnt5a protein; *Wnt5a*^*u−*^, urine-negative Wnt5a protein

## Discussion

An early diagnosis and monitoring of disease activity of SLE patients has an importantly clinical significance for guiding treatments to reduce end-organ damage and achieve remission. By reviewing all available literatures, only one study was found that discussed the role of noncanonical Wnt signaling in the development of SLE [13]. Yet, no studies addressed the role of Wnt5A protein in the early detection of SLE and accessing the severity of SLE patients.

The Wnt5A, a representative ligand that activates noncanonical Wnt signaling in the regulation of cell migration and polarity during embryonic morphogenesis, which has been postulated to be a macrophage effector molecule or to act as a pro-inflammatory factor in macrophages, induce inflammation. Therefore, perturbations in Wnt5A signaling have been reported in various chronic inflammatory/autoimmune rheumatic diseases [10, 21]. For instances, Wnt5A protein and mRNA has been described as being expressed in different inflammatory conditions such as RA, atherosclerosis (ATH), and tuberculosis [10]. It has now become apparent that JAK-STAT3/NF-ҡB/TLR signaling is an important activation mechanism for Wnt5A expression [22, 23]. Moreover, other cell types like lymphocytes, endothelial cells, and smooth muscle cells that are known to express receptor for Wnt5A[23, 24] may mediate paracrine signaling upon Wnt5A-frizzled interactions to trigger an inflammatory response. With respect to ATH, Wnt5a was found to be highly expressed in macrophage-rich regions of both murine and human ATH lesions, respectively [25]. Furthermore, the amount of Wnt5a protein in the serum of ATH patients was significantly higher compared to that of healthy controls [25]. Of great interest, many studies highlighted that SLE is associated with coronary heart disease and ATH [26]. An important prospective study has further demonstrated that SLE patients have an accelerated progression of carotid plaque formation compare to nonlupus controls. Particularly, SLE women, age range 35–44, in respect to healthy subjects have a 50 times increased risk of myocardial infarction and accelerated ATH, that is, a well-recognized comorbidity in SLE [27]. Therefore, the aberrant activation of Wnt5A-mediated noncanonical Wnt signaling may be instrumental in promoting SLE. In the present study, we examined Wnt5A protein in plasmas and urines of SLE patients, and identified that both plasma and urine Wnt5A had a strong association with the severity and progression of SLE patients relative to healthy cohorts. Even higher Wnt5A concentration was observed in the plasmas and urines of AD SLE patients, in comparison with those with LDA SLE patients. Intriguingly, the concentration of Wnt5A protein in SLE patients showed association between plasma and urine. Of note, positive correlations between plasma Wnt5A and dsDNA, urine Wnt5A, and anti-C1q antibody were detected in SLE patients in clinical settings. In agreement with our findings, dsDNA and anti-C1q antibody also were reported to have diagnostic and monitoring values for SLE [28–30]. For instances, an association between SLE and increased dsDNA was detected in a group of 96 SLE patients [31]; such an association was also identified as anti-C1q antibody and SLE [31]. More interestingly, the ROC curve and discriminant function analysis also suggested that the plasma and urine Wnt5A protein could be considered positive biomarkers for the identification of SLE, and severity and/or progression of SLE. Of note, the plasma and urine Wnt5A were separated and distinct only for the AD SLE patients and healthy controls; less reliable separation was found in patients with an LDA pattern of SLE. Given the fact that LDA SLE patients are based on the score of SLEDAI, which focused on new or recurrent manifestations and failed to capture ongoing activity [32], this observation is required to further investigate larger sample sizes.

Apart from the dsDNA and anti-C1q antibody, several serological indexes, such as complements C3 and C4, and immunoglobulin G (IgG) also were reported to have diagnostic and monitoring values for SLE. In this regard, complements C3 and C4 showed high sensitivities and specificities for discrimination between healthy cohorts and SLE patients [33]. In addition, an elevated plasma IgG level was found in SLE patients compared with healthy individuals [34]. However, in contrast to the dsDNA and anti-C1q antibody, neither plasma nor urine Wnt5A protein exhibited a correlation with complements C3 and C4, and IgG in this study. Sample size, different demographics, and treatment with different medicines are thought to contribute to these disparities.

Assessment of multisystem manifestations is the other basis for early diagnosis and assessment of SLE besides laboratory abnormalities ^44^, such as skin rashes, fatigue and weakness, flu-like symptoms, Raynaud’s syndrome (a disorder of the vascular system), increased risk of miscarriage, inflammation of the tissues covering internal organs, neurological problem, kidney problems, oral/nasal ulcers, hair loss, and hematological and psychiatric disorders [35]. Among these, the musculoskeletal system is involved in around 90% of patients with SLE. In addition to myalgia and arthralgia, arthritis of small and large joints may occur [36]. Indeed, about 50% of patients with SLE also have renal involvement [37], who may ultimately develop lupus nephritis (LN), a glomerular nephritis, typically can be characterized with proteinuria and erythrocyturia (particularly dysmorphic erythrocytes), and erythrocyte cylinders in the urinary sediment [38]. Moreover, the central nervous system can also be affected in about 15 to 50% of patients with SLE, but due to the low specificity (e.g., headache) and high variability of the symptoms, their identification as part of SLE often proves difficult [36]. Our findings concurred with their findings as the major affected domains were renal, musculoskeletal, hematological, neuropsychiatric, serositis, and cutaneous in this study.

With respect to serositis, the prevalence of it in SLE varies widely worldwide. In a European study of SLE, the prevalence was 36% [39], while in some Arab countries, the prevalence varied from 15 to 56% [40]. In Hong Kong, the prevalence of SLE-related serositis was 12% [41]. Nevertheless, this condition is not uncommon in SLE patients. Several studies of Chinese SLE patients have indicated that serositis usually correlated with SLE activity and patient survival [40]. In our study, we also found plasma and urine-positive Wnt5A had a stronger association with serositis. Meanwhile, an association also identified cutaneous lesions and SLE; this was further supported by the clinical CLASI activity score which was higher in patients with detectable plasma and urine Wnt5A protein compared to those with negative Wnt5A. In line with our findings, cutaneous manifestations occur in about 75% of patients with SLE in the course of the disease progression, and are the first sign in a quarter of cases [42]. It is important to recognize the different specific cutaneous lesions in SLE (e.g., “butterfly” rash in acute, annular, or psoriasiform photosensitive lesions in the subacute form, and discoid lesions in the chronic form) for an early diagnosis and to estimate the associated risks of internal disease, whereas nonspecific lesions (exanthema, vasculitis, and alopecia) can be part of SLE flares (Fig. 1) [43]. Indeed, forced expression of a secreted Wnt5A in the deeper wound induced changes in the interfollicular epithelium mimicking regeneration in the C57BL mouse wound healing model [44], where Wnt5A was found to lead an invasiveness [45] of the stratified interfollicular epithelium towards the morphogenic gradient with epithelial appendage formation.

In addition, we did not find any correlation between the plasma and urine Wnt5A with the prevalence of renal disorder, musculoskeletal, hematological, or neuropsychiatric involvement in this study. This phenomenon could be explained by numerous and diverse factors, including advanced age, longer disease duration, treatment with different medicines, and the small sample size in each category of organ or system manifestation [46]. In addition to that, the total plasma Wnt5A level may not accurately reflect the overall disease activity as the expression may be localized to the affected tissues only such as cerebrospinal fluid (CSF) and skin [47]. Indeed, over recent years, increasing amount of data on organ damage caused through use of corticosteroids and their cumulative dose has become available. Thus, it has been recommended that of corticosteroids, it should be as low as possible. The same was seen in relation to mycophenolate mofetil and pulse of cyclophosphamide [48]. Given that unlike what is seen in CLASI activity score, no difference was found between SLE patients with plasma- and urine-positive Wnt5A and urine-negative Wnt5A protein (Table 1). One possibility is that the CLASI damage score is based on the degree of scarring and/or atrophy and/or panniculitis, dyspigmentation lesion, and scarring scalp, but a small size of AD SLE samples analyzed in this study therefore is not powered enough to detect any significant difference; this observation is required for further investigate with larger sample sizes.

## Conclusion

In summary, there were several limitations in this study. First, the relatively small number of patients was included in this study, particularly AD SLE patients, which limited our power to detect statistical differences in various clinical manifestations. Second, the outcome data of all enrollments lack follow-up such as a lack of Wnt5A protein testing after receiving standard-of-care therapy. Third, most SLE patients do not undergo surgical skin biopsy to confirm the pathological type. Finally, we were unable to determine the ELISA kit’s precise sensitivity. These limitations may partially explain the discrepancies between our study and other studies. Therefore, these findings require further confirmation in a larger and more selected population in the future.

## Supplementary Information

Below is the link to the electronic supplementary material.

Supplementary file1 (PDF 63 KB)

## Data Availability

The datasets used and/or analyzed during the current study are available from the corresponding author on reasonable request.

## Abbreviations

ACR: American College of Rheumatology
AD: Active disease
ANA: Antinuclear antibodies
AS: Ankylosing spondylitis
ATH: Atherosclerosis
AUC: Area under the curve
CLASI: Cutaneous Lupus Erythematous Disease Area and Severity Index
C3: Complement C3
C4: Complement C4
dsDNA: Double-stranded DNA
ELISA: Enzyme-linked immunosorbent assays
ESR: Erythrocyte sedimentation rate
IgA: Immunoglobulin A
IgG: Immunoglobulin G
IgM: Immunoglobulin M
LDA: Low disease activity
RA: Rheumatoid arthritis
ROC: Receiver operating characteristic
SD: Standard deviation
SEM: Standard error of mean
SLE: Systemic lupus erythematosus
SLEDAI: SLE Disease Activity Index
