# Supplementary material for: Correlation of plasma and urine Wnt5A with the disease activity and cutaneous lesion severity in patients with systemic lupus erythematosus
Source: Immunol Res. 2021 Dec 3;70(2):174–84. doi: 10.1007/s12026-021-09253-w (PMC8917110; doi:10.1007/s12026-021-09253-w)
Supplement: Supplementary file 1 — Supplementary file1 (PDF 63 KB) [file 12026_2021_9253_MOESM1_ESM.pdf]

**SUPPLEMENT INFORMATION**

## Supplemental Tables

### Table S1. Demographic and clinical parameters of SLE patients and healthy cohorts.

| Patient characteristic | Plasma | | | Urine | | |
|---|---|---|---|---|---|---|
| | SLE (n=115) | HC (n=82) | p-value | SLE (n=128) | HC (n=82) | p-value |
| **Demographical features** | | | | | | |
| Ethnics (Chinese Han/Hui) | 89/26 | 68/14 | 0.341 | 88/40 | 60/22 | 0.493 |
| Age (mean±SD)(range, year old) | 40.32±12.11 | 34.56±9.88 | 0.412 | 42.12±11.35 | 33.78±10.12 | 0.434 |
| Gender (male/female) | 35/80 | 22/60 | 0.582 | 47/81 | 30/52 | 0.984 |
| **Disease features** | | | | | | |
| Disease duration (mean±SD) | 10.56±4.12 | | | 11.72±4.48 | | |
| SLEDAI score (range) | 10.66±5.67 | | | 12.78±6.12 | | |
| **Clinical manifestations** | | | | | | |
| Renal disorder (n,%) | 55(47.82%) | | | 70(54.69%) | | |
| Musculoskeletal (n,%) | 50(43.48%) | | | 55(42.97%) | | |
| Haematological (n,%) | 111(96.50%) | | | 119(92.97%) | | |
| Neuropsychiatric (n,%) | 27(23.48%) | | | 29(22.66%) | | |
| Serositis (n,%) | 27(23.48%) | | | 21(16.41%) | | |
| cutaneous (n,%) | 76 (70.40%) | | | 85 (66.41%) | | |
| **Treatment** | | | | | | |
| Prednisone (n,%) | 90(78.26%) | | | 97(75.78%) | | |
| Azathioprine (n,%) | 68(59.13%) | | | 72(56.25%) | | |
| Antimalarial (n,%) | 64(55.65%) | | | 64(50.79%) | | |
| Cydophosphamide (n,%) | 14(12.17%) | | | 21(16.41%) | | |
| Methotrexate (n,%) | 21(18.26%) | | | 25(19.53%) | | |
| **Laboratory** | | | | | | |
| ESR (mm h-1,mean±SD) | 32±2.11 | 27±3.14 | 0.157 | 34±3.22 | 28±2.35 | 0.146 |
| Anti-dsDNA (IU/Ml, mean±SD) | 214.45±103.76 | 90.30±70.17 | 0.000 | 235.17±98.21 | 87.23±67.95 | 0.000 |
| C3 (μg/mL, mean±SD) | 0.48±0.18 | 0.60±0.19 | 0.000 | 0.47±0.21 | 0.47±0.13 | 0.112 |
| C4 (μg/mL, mean±SD) | 0.054±0.02 | 0.057±0.02 | 0.146 | 0.055±0.03 | 0.056±0.02 | 0.135 |
| Proteinuria (>500mg/day, n, %) | 50(39.7%) | | | 61(48.4%) | | |

C3: Complement C3; C4: Complement C4; dsDNA: double-stranded DNA; ESR: erythrocyte sedimentation rate; SLE: Systemic lupus erythematosus; SLEDAI: SLE Disease Activity Index

**Table S2. The Cutaneous Lupus Erythematosus Disease Area and Severity Index (CLASI). Reprinted by permission from the University of Pennsylvania, copyright 2009.**

<table>
<tr><th>Erythema</th><th>Scale/<br>Hypertrophy</th><th colspan="2">Anatomical Location</th><th>Scarring/<br>Atrophy/<br>Panniculitis</th><th>Dyspigmentation</th></tr>
<tr><td>0=absent<br>1=pink faint erythema<br>2=red<br>3=dark red; Purple/violaceous/crusted/hemorrhagic</td><td>0=absent<br>1=scale<br>2=verrucous/ hypertrophic</td><td colspan="2"></td><td>0=absent<br>1=scarring<br>2=severely atrophic scarring or panniculitis</td><td>0=absent<br>1=dyspigmentation</td></tr>
<tr><td></td><td></td><td colspan="2">Scalp</td><td></td><td>See below</td></tr>
<tr><td></td><td></td><td colspan="2">Ears</td><td></td><td></td></tr>
<tr><td></td><td></td><td colspan="2">Nose(incl. malar area)</td><td></td><td></td></tr>
<tr><td></td><td></td><td colspan="2">Rest of face</td><td></td><td></td></tr>
<tr><td></td><td></td><td colspan="2">V-area neck(frontal)</td><td></td><td></td></tr>
<tr><td></td><td></td><td colspan="2">Post. Neck&/or shoulder</td><td></td><td></td></tr>
<tr><td></td><td></td><td colspan="2">Chest</td><td></td><td></td></tr>
<tr><td></td><td></td><td colspan="2">Abdomen</td><td></td><td></td></tr>
<tr><td></td><td></td><td colspan="2">Back, buttocks</td><td></td><td></td></tr>
<tr><td></td><td></td><td colspan="2">Arms</td><td></td><td></td></tr>
<tr><td></td><td></td><td colspan="2">Hands</td><td></td><td></td></tr>
<tr><td></td><td></td><td colspan="2">Legs</td><td></td><td></td></tr>
<tr><td></td><td></td><td colspan="2">Feet</td><td></td><td></td></tr>
<tr><td colspan="2">Mucous membrane lesions (examine if patient confirms involvement)<br>0=absent<br>1=lesion or ulceration</td><td colspan="2"></td><td colspan="2">Dyspigmentation usually lasts less than 12 months (score above remains) or at least 12 months (score is double)</td></tr>
<tr><td colspan="6">Divide the scalp into four quadrants as shown. The dividing line between right and left is the midline. The dividing line between frontal and occipital is the line connecting the highest points of the ear lobe.</td></tr>
<tr><td colspan="2" rowspan="2">Alopecia (clinically not obviously scarred)<br>0=absent<br>1=diffuse; noninflammatory<br>2=focal or patchy in one quadrant<br>3=focal or patchy in one quadrant</td><td>Total Activity Score</td><td>Total Damage Score</td><td colspan="2" rowspan="2">Scarring of the scalp (judged clinically)<br>0=absent<br>1=in one quadrant<br>2=two quadrants<br>3= three quadrants<br>4=affects the whole skull</td></tr>
<tr><td></td><td></td></tr>
</table>
